# Evaluation of a Rapid Point of Care Test for Detecting Acute and Established HIV Infection, and Examining the Role of Study Quality on Diagnostic Accuracy: A Bayesian Meta-Analysis

**DOI:** 10.1371/journal.pone.0149592

**Published:** 2016-02-18

**Authors:** Megan Smallwood, Rohit Vijh, Bénédicte Nauche, Bertrand Lebouché, Lawrence Joseph, Nitika Pant Pai

**Affiliations:** 1 Department of Epidemiology, Biostatistics and Occupational Health, McGill University, Montréal, Quebec, Canada; 2 Division of Clinical Epidemiology, Department of Medicine, McGill University Health Centre, Montreal, Quebec, Canada; 3 Medical Library, Royal Victoria Hospital, McGill University Health Centre, Montreal, Canada; 4 Chronic Viral Illness Service, Research Institute of the McGill University Health Centre, Montreal, Canada; 5 Department of Family Medicine, McGill University, Montreal, Canada; 6 Department of Medicine, McGill University, Montreal, Quebec, Canada; University of Utah Health Sciences Center and ARUP Laboratories, UNITED STATES

## Abstract

**Introduction:**

Fourth generation (Ag/Ab combination) point of care HIV tests like the FDA-approved Determine HIV1/2 Ag/Ab Combo test offer the promise of timely detection of acute HIV infection, relevant in the context of HIV control. However, a synthesis of their performance has not yet been done. In this meta-analysis we not only assessed device performance but also evaluated the role of study quality on diagnostic accuracy.

**Methods:**

Two independent reviewers searched seven databases, including conferences and bibliographies, and independently extracted data from 17 studies. Study quality was assessed with QUADAS-2. Data on sensitivity and specificity (overall, antigen, and antibody) were pooled using a Bayesian hierarchical random effects meta-analysis model. Subgroups were analyzed by blood samples (serum/plasma vs. whole blood) and study designs (case-control vs. cross-sectional).

**Results:**

The overall specificity of the Determine Combo test was 99.1%, 95% credible interval (CrI) [97.3–99.8]. The overall pooled sensitivity for the device was at 88.5%, 95% [80.1–93.4]. When the components of the test were analyzed separately, the pooled specificities were 99.7%, 95% CrI [96.8–100] and 99.6%, 95% CrI [99.0–99.8], for the antigen and antibody components, respectively. Pooled sensitivity of the antibody component was 97.3%, 95% CrI [60.7–99.9], and pooled sensitivity for the antigen component was found to be 12.3%, 95% (CrI) [1.1–44.2]. No significant differences were found between subgroups by blood sample or study design. However, it was noted that many studies restricted their study sample to p24 antigen or RNA positive specimens, which may have led to underestimation of overall test performance. Detection bias, selection (spectrum) bias, incorporation bias, and verification bias impaired study quality.

**Conclusions:**

Although the specificity of all test components was high, antigenic sensitivity will merit from an improvement. Besides the accuracy of the device itself, study quality, also impacts the performance of the test. These factors must be kept in mind in future evaluations of an improved device, relevant for global scale up and implementation.

## Introduction

In 2014, an estimated 36.9 million people were living with HIV worldwide; with a vast majority residing in low and middle-income countries. [1] While advances in antiretroviral therapy (ART) have greatly reduced the morbidity and mortality rates from HIV, rising new infection rates remain a concern, with 2.1 million new infections occurring in 2013. [2] The risk of HIV transmission is highest during acute and early infection, accounting for up to 50% of new infections [3–7].

Early identification of HIV infection and timely knowledge of HIV status is crucial for improving treatment initiation, and potentially reducing disease transmission to sexual partners. [8, 9] Advantages to beginning ART early include improved CD4 counts, suppression of viral load, and control of HIV infection in the community. [10–12] In response to recent evidence, the WHO has issued recommendations for earlier initiation of ART, to increase the quality and duration of the lives of those with HIV. [13] The challenge remains in identifying people with acute and early HIV infection, and bringing them into care early on.

Antibody based rapid and point of care (POC) tests for HIV have allowed expanded access to HIV testing and have been a breakthrough for HIV testing in resource-limited settings, and high income settings alike. [14–18] Patients can receive rapid results, saving time and repeat visits. [19] However, the second and third generations of rapid HIV POC tests are antibody based, inherently limited to detecting HIV infection post-seroconversion (after 3–4 weeks). [6] These tests cannot detect infection in the acute phase “window period”. [20] This acute phase is challenging for HIV diagnosis since antibodies are not yet detectable in the blood, however viral RNA is present (as of 10 days post exposure), and another marker, p24 antigen, becomes detectable 15–17 days post infection. [21] In comparison, HIV antibodies are detected in approximately 20–22 days post infection (5 days after p24 antigen). [22] While third generation rapid tests perform well for detecting HIV antibodies (sensitivity > 95%, specificity > 99%), [19, 23] newer fourth generation laboratory-based tests claim to detect HIV approximately 5 days earlier than third generation tests by detecting p24 antigen in addition to antibodies. [6, 24] Therefore, to detect early HIV infection, these tests would be a welcome addition to the repertoire of rapid POC tests, if found to be accurate.

Recently, the U.S. Centers for Disease Control and Prevention (CDC) has issued updated laboratory recommendations to initially test for suspected HIV with a fourth generation antigen-antibody assay, and has called to replace dated antibody-only based tests. [25] The vast majority of fourth generation assays are laboratory based and are not suitable for use at the point of care (POC), but recently, one rapid fourth generation assay was approved by the FDA to aid in the rapid diagnosis of acute HIV infection: the Determine HIV-1/2 Combo Ag/Ab Rapid test (Alere Inc., Waltham, MA). [26] The Determine Combo test is a qualitative, point of care test, which provides results within 20 minutes and is able to distinguish p24 antigen and HIV antibody results. [27] Due to insufficient information on its field performance, the CDC has yet to recommend the use of the Determine Combo test in place of 4^th^ generation laboratory tests, hence the need for a systematic retrieval and synthesis of diagnostic performance. [25] To the best of our knowledge, a critical review of its field performance has not yet been done.

### Objectives

In this context, we aim to synthesize evidence the diagnostic performance (accuracy parameters) of the Determine Combo test. Additionally, we will explore the effects of various study designs, and blood sample type on the estimates of accuracy. We will compare the pooled sensitivity and specificity of antigen and antibody components, separately.

## Methods

### Data Sources and Searches

Preferred Reporting Items for Systematic Reviews and Meta-Analyses (PRISMA) guidelines were followed for reporting/conducting the review. [28] A systematic search of the literature was conducted by a librarian (BN) to retrieve all studies which evaluated the Determine Combo test. A search strategy was developed for Medline via OvidSP, and then adapted to other databases: Embase, Biosis Previews, The Cochrane Library (including CENTRAL), PubMed (limited to records “as supplied by publisher”), LILACS, and African Index Medicus.

The search strategies were designed to retrieve the Determine Combo test and synonyms in all appropriate fields, and a combination of MeSH terms and text words in appropriate fields to capture acute, early and incident HIV infection. No publication year limit was used. The searches were run June 27, 2014, and updated on January 7^th^ 2015. Relevant conference abstracts, bibliographies in relevant primary studies, narrative reviews, editorials, and citations from selected studies were hand-searched. Abstracts were included if they provided sufficient information. Both English and Non-English articles were included. (Please refer to the S1 file for search strategies details).

### Study Selection and eligibility criteria

Two independent reviewers performed the first screen (MS and RV). Studies were included if they met the following eligibility criteria: evaluated the Determine Combo Test against a reference standard in adult populations, and reported effect measures such as sensitivity and specificity (or raw cell values such as true positives, true negatives, false positives, false negatives). Study inclusion was un-restricted by country, study design, or patient populations.

Studies were excluded if the Determine Combo test was evaluated in children or infants only, or if the reference standard was not performed on all samples.

Full-text articles were retrieved, and assessed for eligibility by two independent reviewers (MS and RV). Disagreements were resolved through discussion, and consultation of a third reviewer (NPP).

### Data Extraction and Quality Assessment

Two reviewers (MS and RV) independently extracted data and performed quality assessment for each study using the QUADAS-2 tool (Quality Assessment of Diagnostic Accuracy Studies). [29] QUADAS-2 consists of four domains: patient selection, index test, reference standard, and flow and timing. Quality is assessed in each domain in terms of risk of bias, and concerns regarding applicability. The patient selection domain assesses whether or not the way patients were selected or excluded from study participation could have introduced bias into the study. [29] The index test and reference standard domains assess whether the conduct or interpretation of the index test or reference standard, respectively, may have introduced bias. [29] The flow and timing domain addresses the time interval between index test and reference standard, and if all patients received the same reference standard [29].

The data abstraction form contained the following variables: authors, year of publication, objectives, study design, country, patient population, HIV prevalence in sample, total sample size, conflict of interest, reference standards, blood sample type, proportion of antigen positive subjects in sample, outcomes reported, sensitivity and specificity (overall, and antibody and antigen components separately), and raw cell values (true positives, false positives, true negatives, false negatives) if provided.

Disagreements between reviewers (MS and RV) were resolved by consensus and in consultation with a third reviewer (NPP).

### Data Synthesis and Analysis

Many different reference standard testing algorithms were used across studies, with varying initial screening and confirmatory testing, (please see S1 Table), however due to data limitations we were unable to analyze the impact of imperfect reference standards. In this analysis we treated each reference standard as a gold standard, and sensitivity and specificity were analyzed in separate models. For the sensitivity, the data consisted of the number of subjects correctly detected as positive (“successes” for positive subjects) by the test out of all subjects classified as truly positive by the gold standard. Similarly, for specificity, the data consisted of the number of subjects correctly detected as negative (“successes” for negative subjects) by the test out of all subjects classified as truly negative by the gold standard.

We used a hierarchical Bayesian random effects meta-analysis model to synthesize results for the sensitivity and specificity of the test. At the first level of our hierarchical model, we assumed that the number of successes from each study included in the meta-analysis followed a binomial distribution, with probability of success parameter allowed to vary by study. At the second level of the hierarchical model we assumed that the logit transformed binomial parameters from the first level followed a normal density, with mean representing the average success rate across all studies, and variance indicating the study-to-study variability in success rates between studies. To complete the Bayesian model, very wide prior densities were used for the mean and variance of the normal density at the second hierarchical level, meaning that the data will drive the final inferences rather than any prior information. We ran analysis for all studies combined, as well as subsets of the studies by study design (case-control vs. cross-sectional) and blood sample type, as we pre-specified. We ran further subgroup analyses by antibody or antigen components.

All models were run in WinBUGS (version 1.4.3, MRC Biostatistics Unit, Cambridge UK). We created forest plots to visually represent individual study results with their confidence intervals using Review Manager (RevMan Version 5.3. Copenhagen: The Nordic Cochrane Centre, The Cochrane Collaboration, 2011).

## Results

A total of 17 studies that reported diagnostic accuracy of the Determine Combo test were included in the final selection. [30–46]

**Fig 1:**
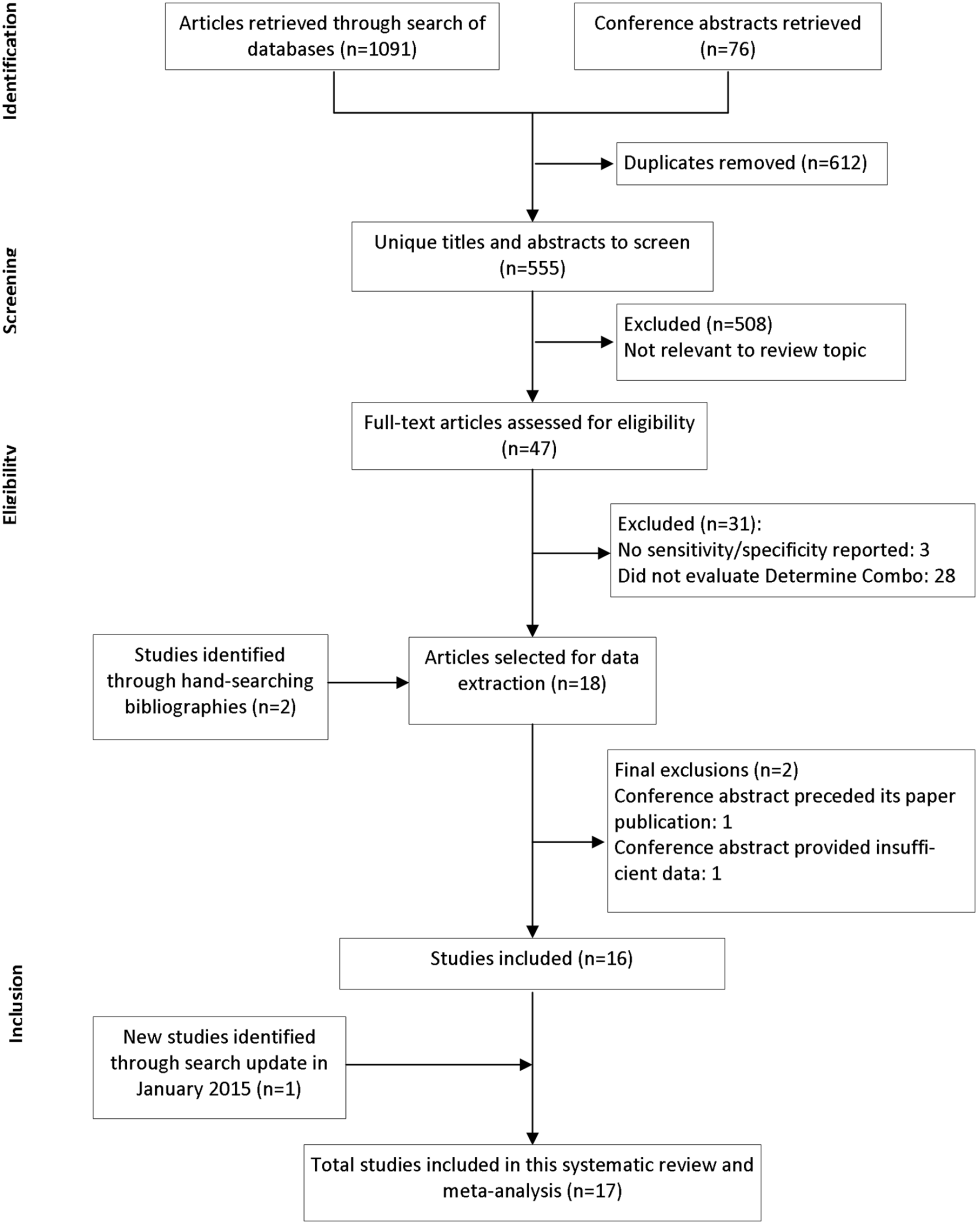
PRISMA flow diagram of study selection. The flow diagram can be broken down by stage, beginning at identification of records, screening titles and abstract, screening full-text for eligibility, and study inclusion.

A flow diagram illustrates the number of records obtained during the process of study selection (Fig 1). The demographic characteristics of all included studies are in S1 Table.

**Fig 2:**
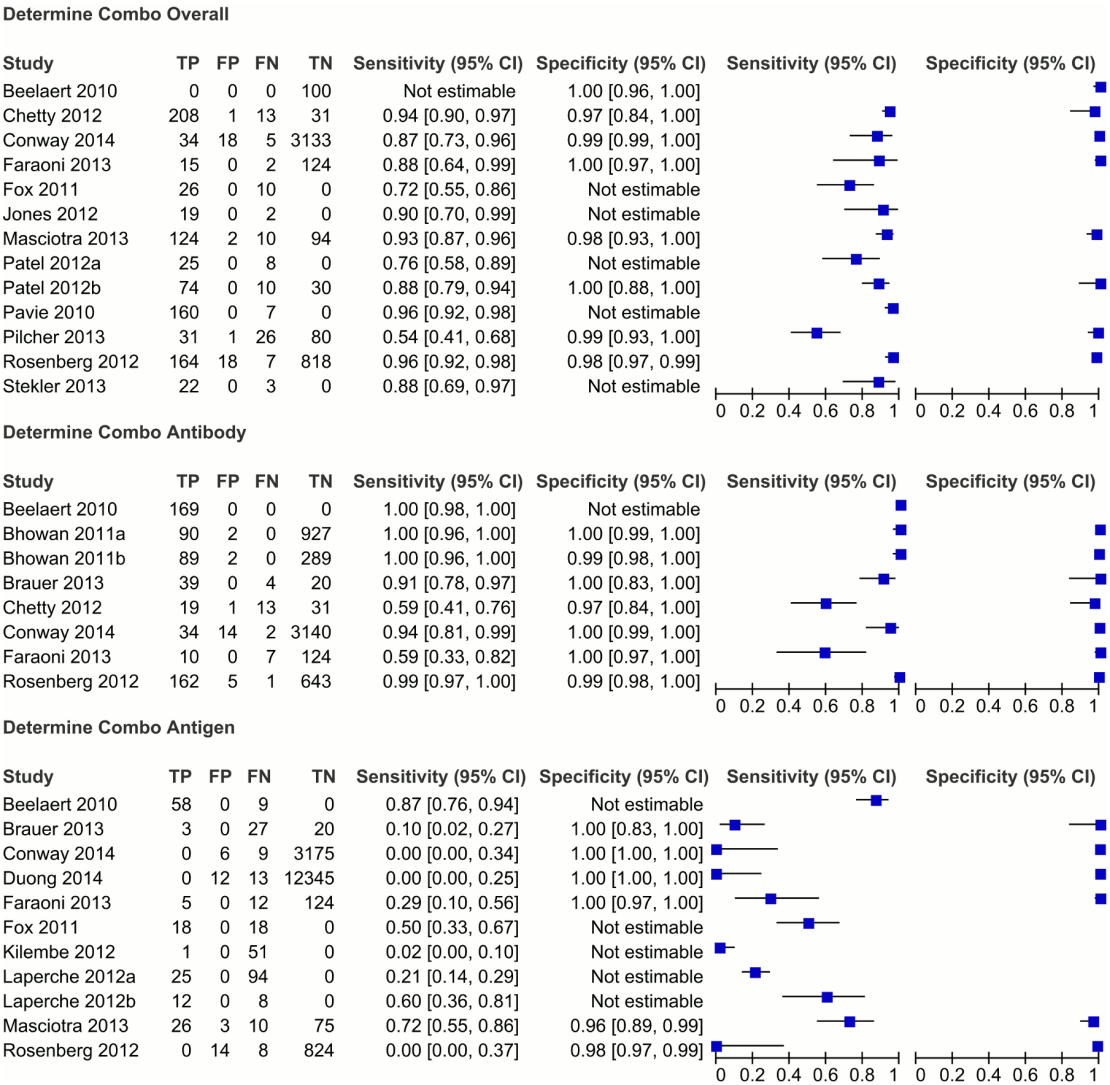
Forest plots showing performance of the Determine Combo test in individual studies. The first forest plot shows the studies which evaluated the overall performance of the test, the second plot shows studies which evaluated the antibody component, and the third plot shows studies which evaluated the antigen component. The blue squares represent the estimate for sensitivity or specificity from each study, and the horizontal lines represent the 95% confidence intervals. It should be noted that missing data (for TP, FP, FN, TN) was treated as having a value of zero by RevMan software; this did not affect the sensitivity and specificity estimates, however please refer to S2 Table for raw cell values extracted from each study.

Ten studies used a case-control design, and seven were cross-sectional. Whole blood samples were collected in seven studies, with either plasma or serum samples collected in the remaining ten studies. Three studies (Patel et al, Laperche et al, and Bhowan et al) each contained more than one separate, unique evaluation of the Determine Combo test, and in these cases each evaluation was treated independently. [31, 39, 41] Individual study estimates with their confidence intervals are represented through forest plots as seen in Fig 2. Raw data extracted from each individual study can be seen in S2 Table.

### Primary Finding

The overall pooled sensitivity estimate for the assay was 88.5%, 95% CrI [80.1–93.4], and overall pooled specificity was 99.1%, 95% CrI [97.3–99.8].

When the individual antigen and antibody components of the assay were evaluated separately, the antigen component produced a pooled sensitivity of 12.3%, 95% CrI [1.1–44.2], with a pooled specificity of 99.7%, 95% CrI [96.8–100].

The antibody component produced a pooled sensitivity of 97.3%, 95% CrI [60.7–99.9], and pooled specificity of 99.6%, 95% CrI [99.0–99.8].

Results estimating sensitivity and specificity from our Bayesian hierarchical meta-analytical models are given in Table 1.

**Table 1:** Results from Bayesian hierarchical meta-analysis. Point estimates given are posterior medians, with 95% credible intervals (CrI).

**Overall**	**Sensitivity**	**95% CrI**	**Specificity**	**95% CrI**
**Total**	88.5	80.1–93.8	99.1	97.3–99.8
**Serum**	84.3	65.0–94.2	99.3	94.7–100.0
**Whole blood**	93.8	84.4–97.3	98.8	0.00–100.0
**Case-control**	84.9	65.0–94.5	99.6	79.1–100.0
**Cross-sectional**	93.2	83.9–96.9	98.8	37.2–100.0
**Antibody**	**Sensitivity**	**95% CrI**	**Specificity**	**95% CrI**
**Total**	97.3	60.7–99.9	99.6	99.0–99.8
**Serum**	*	*	99.8	83.9–100.0
**Whole blood**	*	*	99.5	93.4–99.9
**Case-control**	*	*	*	*
**Cross-sectional**	99.2	35.6–100.0	99.5	98.4–99.8
**Antigen**	**Sensitivity**	**95% CrI**	**Specificity**	**95% CrI**
**Total**	12.3	1.1–44.2	99.7	96.8–100.0
**Serum**	27.8	5.9–67.4	*	*
**Whole blood**	*	*	99.7	24.7–100.0
**Case-control**	27.8	5.9–67.4	*	*
**Cross-sectional**	*	*	99.7	24.7–100.0

* These parameters could not be estimated due to limited data

To compare the diagnostic accuracy of the Determine Combo test in serum/plasma vs. whole blood samples, we performed a subgroup analysis. We found the overall pooled sensitivity in serum/plasma to be 84.3%, 95% CrI [65.0–94.2], and specificity to be 99.3%, 95% CrI [94.7–100.0]. The overall pooled sensitivity in whole blood was 93.8%, 95% CrI [84.4–97.3]. An estimate for pooled specificity in whole blood could not be provided by the data available.

Overall diagnostic accuracy was also assessed in subgroups by study design, with case-control studies reporting a pooled sensitivity of 84.9, 95% CrI [65.0–94.5] and pooled specificity of 99.6, 95% CrI [79.1–100.0]. Cross-sectional studies had a pooled sensitivity of 93.2%, 95% CrI [83.9–96.9], and specificity of 98.8, 95% CrI [37.2–100.0].

**Fig 3:**
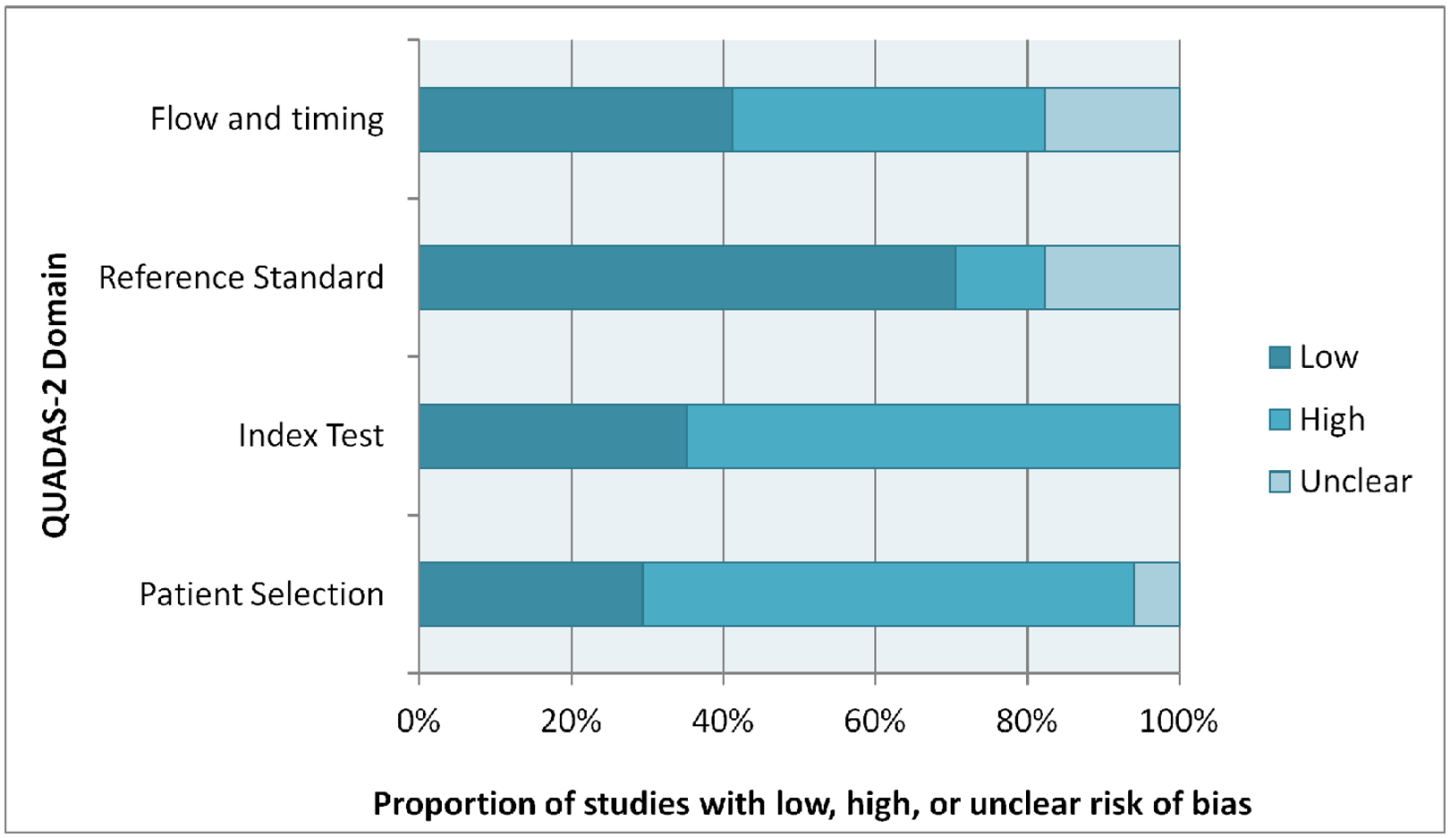
Graphical representation of quality assessment using QUADAS-2. Each domain of QUADAS-2 is represented by a horizontal bar, divided into the proportion of studies which scored low, high, or unclear risk of bias.

Quality assessment was conducted for each study with the use of the QUADAS-2 tool; please refer to the summary presented in Fig 3.

## Discussion

This meta-analysis identified 17 studies evaluating the diagnostic performance and accuracy of the Determine Combo test, across various global settings. In our meta-analysis, we found the antibody component had a high pooled sensitivity (97.3%, 95% CrI 60.7–99.9) for HIV antibodies, however the antigen component was found to have very low sensitivity (12.3%, 95% CrI 1.1–44.2) for p24 antigen.

The manufacturers of this assay claimed an overall sensitivity of 100% for the Combo test, and specificities of 99.23% and 99.66% for the antibody and antigen components, respectively. [47] We found the pooled specificity estimates in this meta-analysis (antibody specificity 99.6%, antigen specificity 99.5%) to be consistent with the performance claims, however the overall pooled sensitivity result (overall sensitivity 88.5%, 95% CrI [80.1–93.4]) was not consistent with claims of 100% sensitivity.

With respect to our secondary findings, for both subgroups by blood sample type or by study designs, there were no substantive differences in sensitivity or specificity. Studies using whole blood had a higher pooled sensitivity and lower specificity than serum/plasma, and cross-sectional studies had a higher pooled sensitivity and lower specificity than case-control studies (understandably, because of the patient spectrum recruited in these studies), however these estimates were imprecise due to small sample sizes (all credible intervals greatly overlap). Although in one recent CDC field study with the largest sample size (N = 18 172), the data largely parallels our findings [46].

Quality assessment of studies in this meta-analysis points to the need to design studies of diagnostic accuracy with careful attention to methodology. Many studies included in this meta-analysis were of low methodological quality, particularly in the “index test” and “patient selection” domains, (64.7% of studies) as outlined by the QUADAS-2 tool. This was largely due to case-control study designs which tend to be less representative of real patient populations than cross-sectional designs [29].

Many studies were assigned a high risk of bias due to lack of blinding of the reference test results when interpreting the index test, which is a critical step in preventing detection bias—only 29.4% of studies clearly described blinding of reference standard results. Studies which selected patients or samples into the study based on their HIV status (i.e. case-control designs) were also assigned a high risk of bias, as this type of patient selection tends to overestimate test accuracy by selecting patients with more obvious or extreme symptoms. Case-control studies often select well-characterized samples (those which are clearly positive or clearly negative) and exclude samples which produce unclear results, rather than selecting samples which are representative of the broad spectrum of disease. This puts the study at risk of bias, known as spectrum bias.[48] Studies were also at risk of incorporation bias and verification bias by not using the same reference standard for all samples. In some cases, the results of the index test were used to determine which reference standard was used; in other cases, the index test was performed again if the results differed from the reference standard.

The results of the study quality assessment should be kept in mind when interpreting the pooled meta-analysis estimates. As seen in Fig 3, 65% of studies reported high risk of bias in the “patient selection” domain, and 65% of studies reported high risk of bias in the “index test” domain. 13 out of 17 studies (75%) scored a “high” risk of bias in two QUADAS-2 domains or more, and not a single study scored a “low” risk of bias in all domains.

There are many reasons as to why the antigen component of the Determine Combo test performs poorly. One potential explanation may be that the Determine Combo test lacks heat dissociation and signal amplification steps, which are not feasible for rapid testing but have been shown to improve the detection of p24 antigen. [44] Other explanations include nonspecific binding of p24 antigen, and the limit of detection for p24 antigen may not be adequately low. [44, 46] The p24 antigen sensitivity may also have been underestimated in this meta-analysis if p24 antigen was evaluated in samples that were restricted to “acute” defined as antibody negative and RNA positive. There is a delay of five days between RNA and p24 antigen detection; so unless a reference standard was used which was able to independently detect p24 antigen, the accuracy of this component may be underestimated. The performance of the antigen component would also be underestimated if evaluated in samples which had already seroconverted (antibody positive but antigen negative), however we did not include these types of estimates in the meta-analysis. The discrepancies between the two components of the test explain why the Determine Combo test performs similarly to third generation tests (which are only able to detect HIV antibodies), but cannot perform at the same level as fourth generation lab tests which are much more sensitive for detecting early infection.

Multiple studies concluded that the performance of the Determine test was poor for detecting acute HIV, [31, 36–39, 44, 46] and this meta-analysis provides evidence that the Determine Combo test, in its present form, is more suitable for use in testing/screening those populations which have already seroconverted. It should be noted that the accuracy of this test independent of the antibody component is only relevant for the period preceding seroconversion (the acute phase), when an antibody test would be negative. Even if the antigen sensitivity is low, it may still be an improvement on third generation tests; however, in settings with access to advanced laboratory equipment, a fourth generation laboratory test may be more useful for detecting acute infections.

These findings should be interpreted with certain caveats.

Firstly, while not analyzed statistically, descriptively, it can be seen that the overall sensitivity reported by each individual study varied based on the patient spectrum (case mix). For example, in order to increase the sample size for antigen positive subjects, some studies restricted their analyses to samples of acute or recent infections only (antigen or RNA positive, but western blot negative). Since antibodies are not detectable in the early stages of infection, the overall performance of the test will be disproportionally weighted to the antigen component, which has a very different performance. This trend seems to suggest that the overall sensitivity of the Determine Combo test might depend on the prevalence of acute specimens in the study sample. If the study sample consists of both recent and established infections (mirroring the real-life spectrum of patients that show up for testing), the overall sensitivity of the test will likely be higher than a study sample restricted to acute patients.

Secondly, the role of HIV-1 Non B subtypes and HIV-2 in predicting a poor performance of this test remains unknown. HIV-1 Non B subtypes infection (Subtype C, D, E) may impair performance of a p24 antigen test [30, 44].

Thirdly, the CDC recommends initial testing with a fourth generation test, followed by confirmatory antibody testing and NAAT if these results are discrepant. [25] If we consider this algorithm as the gold standard, then only one of the 16 studies included in this review had a perfect gold standard (Masciotra et al), [40] with many studies initially testing using third generation assays, with little to no consistency between studies. Comparison with an inferior assay, such as a third generation assay or rapid test, underestimates performance parameters (i.e., sensitivity and specificity). On the other hand, if RNA is used as the gold standard for p24 antigen sensitivity, the estimate may be biased due to RNA positive samples that are not yet antigen positive [49].

Future studies evaluating the separate components of the Determine Combo test should use a reference standard that is able to accurately identify p24 antigen, such as a fourth generation immunoassay or p24 assay. In addition, future studies should be mindful of biases that we observed and take note of the spectrum of patients in their study sample.

Strengths: include a broad and extensive literature search, independent data extraction and study quality assessment, Bayesian hierarchical random effects methods for meta-analysis, and sub-group analysis by study design and blood samples.

Limitations: Possible publication bias, as statistical tests and funnel plots to formally assess publication bias are not recommended anymore. [50] We were also unable to statistically explore the effect of patient case mix and varying references standards.

## Conclusion

To conclude, this is the first Bayesian meta-analysis to synthesize evidence on the overall global performance of the Determine HIV Ag/Ab Combo rapid test. In general, the quality of studies must be improved for evaluating the Determine Combo test, and any other future rapid tests for detecting acute HIV infections.

We found that the Determine Combo test was accurate in identifying HIV antibodies, but based on the studies which evaluated p24 antigen independently of antibodies, its antigen component should be improved to rival fourth generation laboratory assays. An improved antigen component will also make the Determine Combo test the most accurate and useful point-of-care test, better than many antibody based assays. This assay in its current form will be most suitable for identifying HIV infections in those who have seroconverted, but falls short in picking up acute HIV infections.

In resource-rich settings, confirmatory lab based fourth generation laboratory assays and NAAT can detect acute infections, but in resource-limited settings an improved version of the Determine Combo test will potentially replace third generation rapid testing, thereby offering strong competition to the available repertoire of antibody based tests.

## Supporting Information

S1 File Appendix. Complete search string.
(DOC)

S2 File PRISMA 2009 checklist. (DOC)

S1 Table Demographic characteristics of included studies. (DOC)

S2 Table Raw data included in analysis. (DOC)
