# Supplementary material for: Evaluation of a Rapid Point of Care Test for Detecting Acute and Established HIV Infection, and Examining the Role of Study Quality on Diagnostic Accuracy: A Bayesian Meta-Analysis
Source: PLoS One. 2016 Feb 18;11(2):e0149592. doi: 10.1371/journal.pone.0149592 (PMC4758636; doi:10.1371/journal.pone.0149592)
Supplement: S1 File — Complete search string. (DOC) [file pone.0149592.s001.doc]

**Appendix**

**Complete Search String**

# Detailed count of records per database

|  | **Results** | **Duplicates deleted** | **Unique results to screen** |
| --- | --- | --- | --- |
| **Medline articles** | 374 | 1 | 373 |
| **Embase articles** | 404 | 347 | 57 |
| **Embase conferences** | 65 | 1 | 64 |
| **Biosis articles** | 255 | 242 | 13 |
| **Biosis conferences** | 11 | 0 | 11 |
| **Cochrane** | 33 | 21 | 12 |
| **Pubmed** | 3 | 0 | 3 |
| **AIM** | 0 | 0 | 0 |
| **LILACS** | 22 | 0 | 22 |
| **TOTAL** | 1167 | 612 | 555 |

Total records identified through Medline and Embase search update on January 7^th^, 2015: **55**

# Ovid Medline

Database: Ovid MEDLINE(R) In-Process & Other Non-Indexed Citations, Ovid MEDLINE(R) Daily, Ovid MEDLINE(R) and Ovid OLDMEDLINE(R) <1946 to Present>

Search Strategy:

--------------------------------------------------------------------------------

1 ((Alere or inverness) and determine*).tw,kf,ot,oa. (34)

2 (((ag adj2 ab) or (antigen* adj2 antibod*)) adj4 determine*).tw,kf,ot,oa. (167)

3 (Determine* adj4 (HIV* or kit or combo)).tw,kf,ot,oa. (4224)

4 (Determine* adj4 ((4th or "4" or fourth) adj2 generation)).tw,kf,ot,oa. (12)

5 or/1-4 (4413)

6 exp HIV/ or exp HIV infections/ or HIV Antibodies/ or exp HIV Antigens/ (266037)

7 hiv*.mp,jw,in. (279924)

8 (acquired adj2 immun* adj2 syndrome*).mp,jw,in. (93499)

9 (acquired immun* adj3 deficien*).mp,jw,in. (13318)

10 (human immunedeficien* or human immunodeficien* or human immunideficien*).mp,jw,in. (79379)

11 or/6-10 (330779)

12 Acute disease/ (182858)

13 Early diagnosis/ (13258)

14 (acute* adj5 (infect* or disease)).tw,kf. (69445)

15 ((recent* or earl* or primary) adj2 (infect* or disease)).tw,kf. (62739)

16 (immediately adj2 (post or after or following) adj2 (infect* or acquisit* or transmit*)).tw,kf. (616)

17 (AHI or AHIs or PHI or PHIs).tw,kf. (21194)

18 (preseroconver* or pre-seroconver* or ((recent* or primary or new* or initial* or preced* or before) adj2 (seroconver* or sero-conver*)) or ((acquisition* or infection*) adj6 (seroconver* or sero-conver*))).tw,kf. (1896)

19 ((acute or initial or ramp-up) adj2 (phase* or period* or stage*)).tw,kf. (76918)

20 (newly adj2 infected).tw,kf. (764)

21 incident infection*.tw,kf. (257)

22 or/12-21 (395591)

23 11 and 22 (12936)

24 acute retroviral syndrome.tw,kf. (79)

25 incident hiv*.tw,kf. (245)

26 (Acute adj4 (hiv* or ((acquired or human) adj2 immun* adj3 (syndrome* or deficien*)) or human immunedeficien* or human immunodeficien* or human immunideficien*)).tw,kf. (2071)

27 ((recent* or earl* or primary) adj2 (hiv* or ((acquired or human) adj2 immun* adj3 (syndrome* or deficien*)) or human immunedeficien* or human immunodeficien* or human immunideficien*)).tw,kf. (5767)

28 24 or 25 or 26 or 27 (7709)

29 23 or 28 (16904)

30 5 and 29 (406)

31 limit 30 to humans (375)

32 limit 30 to animals (21)

33 30 not (31 or 32) (25)

34 31 or 33 (400)

35 remove duplicates from 34 (374)

# Embase

Database: Embase Classic+Embase <1947 to 2014 June 26>

Search Strategy:

--------------------------------------------------------------------------------

1 Determine*.dv. (159)

2 ((Alere or inverness) and determine*).tw,kw,ot. (101)

3 (((ag adj2 ab) or (antigen* adj2 antibod*)) adj4 determine*).tw,kw,ot. (220)

4 (Determine* adj4 (HIV* or kit or combo)).tw,kw,ot. (5056)

5 (Determine* adj4 ((4th or "4" or fourth) adj2 generation)).tw,kw,ot. (21)

6 or/1-5 (5469)

7 exp Human immunodeficiency virus/ (133977)

8 exp Human immunodeficiency virus infection/ (298910)

9 Human immunodeficiency virus antibody/ (7772)

10 Human immunodeficiency virus antigen/ (1602)

11 exp acquired immune deficiency syndrome/ (127945)

12 hiv*.mp,jw,in. (284514)

13 (acquired adj2 immun* adj2 syndrome*).mp,jw,in. (135406)

14 (acquired immun* adj3 deficien*).mp,jw,in. (130685)

15 (human adj2 immun* adj2 deficien*).mp,jw,in. (766)

16 (human immunedeficien* or human immunodeficien* or human immunideficien*).mp,jw,in. (318983)

17 or/7-16 (411200)

18 Acute disease/ (87850)

19 early diagnosis/ (77122)

20 (acute* adj5 (infect* or disease)).tw,kw. (94775)

21 ((recent* or earl* or primary) adj2 (infect* or disease)).tw,kw. (85558)

22 (immediately adj1 (post or after or following) adj2 (infect* or acquisit* or transmit*)).tw,kw. (767)

23 (AHI or AHIs or PHI or PHIs).tw,kw. (15477)

24 (preseroconver* or pre-seroconver* or ((recent* or primary or new* or initial* or preced* or before) adj2 (seroconver* or sero-conver*)) or ((acquisition* or infection*) adj6 (seroconver* or sero-conver*))).tw,kw. (2215)

25 ((acute or initial or ramp-up) adj2 (phase* or period* or stage*)).tw,kw. (105342)

26 (newly adj2 infected).tw,kw. (860)

27 incident infection*.tw,kw. (321)

28 or/18-27 (440105)

29 17 and 28 (16375)

30 acute retroviral syndrome.tw,kw. (106)

31 incident hiv*.tw,kw. (263)

32 (Acute adj4 (hiv* or ((acquired or human) adj2 immun* adj3 (syndrome* or deficien*)) or human immunedeficien* or human immunodeficien* or human immunideficien*)).tw,kw. (2535)

33 ((recent* or earl* or primary) adj2 (hiv* or ((acquired or human) adj2 immun* adj3 (syndrome* or deficien*)) or human immunedeficien* or human immunodeficien* or human immunideficien*)).tw,kw. (6529)

34 or/30-33 (8866)

35 29 or 34 (20931)

36 6 and 35 (477)

37 limit 36 to human (432)

38 limit 36 to animal (7)

39 36 not (37 or 38) (38)

40 37 or 39 (470)

41 remove duplicates from 40 (469)

42 limit 41 to conference abstract (65)

43 41 not 42 (404)

# Cochrane Library

ID Search Hits

#1 ((Alere or inverness) and determine*):ti,ab 3

#2 (((ag near/2 ab) or (antigen* near/2 antibod*)) near/4 determine*):ti,ab 5

#3 (Determine* near/4 (HIV* or kit or combo)):ti,ab 197

#4 (Determine* near/4 ((4th or "4" or fourth) near/2 generation)):ti,ab 0

#5 {or #1-#4} 203

#6 [mh HIV] or [mh "HIV infections"] or [mh ^"HIV Antibodies"] or [mh "HIV Antigens"] 8091

#7 hiv*:ti,ab 9980

#8 (acquired near/2 immun* near/2 syndrome*):ti,ab 480

#9 (acquired immun* near/3 deficien*):ti,ab 128

#10 (human near/2 immun* near/2 deficien*):ti,ab 14

#11 (human immunedeficien* or human immunodeficien* or human immunideficien*):ti,ab 2594

#12 {or #6-#11} 11750

#13 [mh ^"Acute disease"] 8902

#14 [mh ^"Early Diagnosis"] 415

#15 (acute* near/5 (infect* or disease)):ti,ab 3596

#16 ((recent* or earl* or primary) near/2 (infect* or disease)):ti,ab 1963

#17 (immediately near/2 (post or after or following) near/2 (infect* or acquisit* or transmit*)):ti,ab 8

#18 (AHI or AHIs or PHI or PHIs):ti,ab 612

#19 (preseroconver* or pre-seroconver* or ((recent* or primary or new* or initial* or preced* or before) near/2 (seroconver* or sero-conver*)) or ((acquisition* or infection*) near/6 (seroconver* or sero-conver*))):ti,ab 62

#20 ((acute or initial or ramp-up) near/2 (phase* or period* or stage*)):ti,ab 4072

#21 (newly near/2 infected):ti,ab 9

#22 incident infection*:ti,ab 256

#23 {or #13-#22} 18446

#24 #12 and #23 437

#25 (Acute near/4 (hiv* or ((acquired or human) near/2 immun* near/3 (syndrome* or deficien*)) or human immunedeficien* or human immunodeficien* or human immunideficien*)):ti,ab 52

#26 ((recent* or earl* or primary) near/2 (hiv* or ((acquired or human) near/2 immun* near/3 (syndrome* or deficien*)) or human immunedeficien* or human immunodeficien* or human immunideficien*)):ti,ab 229

#27 acute retroviral syndrome:ti,ab 5

#28 incident hiv*:ti,ab 155

#29 {or #24-#28} 631

#30 #5 and #29 33 184

# Biosis Previews

Database: BIOSIS Previews <1969 to 2014 Week 30>

Search Strategy:

--------------------------------------------------------------------------------

1 ((Alere or inverness) and determine*).tw. (16)

2 (((ag adj2 ab) or (antigen* adj2 antibod*)) adj4 determine*).tw. (147)

3 (Determine* adj4 (HIV* or kit or combo)).tw. (3398)

4 (Determine* adj4 ((4th or "4" or fourth) adj2 generation)).tw. (10)

5 or/1-4 (3559)

6 hiv*.tw,jx,in. (235128)

7 (acquired adj2 immun* adj2 syndrome*).tw,jx,in. (98424)

8 (acquired immun* adj3 deficien*).tw,jx,in. (17116)

9 (human immunedeficien* or human immunodeficien* or human immunideficien*).tw,jx,in. (235244)

10 (human adj2 immun* adj2 deficien*).tw,jx,in. (1474)

11 or/6-10 (294382)

12 (acute* adj5 (infect* or disease)).tw. (165532)

13 ((recent* or earl* or primary) adj2 (infect* or disease)).tw. (49952)

14 (immediately adj2 (post or after or following) adj2 (infect* or acquisit* or transmit*)).tw. (589)

15 (AHI or AHIs or PHI or PHIs).tw. (23092)

16 (preseroconver* or pre-seroconver* or ((recent* or primary or new* or initial* or preced* or before) adj2 (seroconver* or sero-conver*)) or ((acquisition* or infection*) adj6 (seroconver* or sero-conver*))).tw. (1849)

17 ((acute or initial or ramp-up) adj2 (phase* or period* or stage*)).tw. (67799)

18 (newly adj2 infected).tw. (624)

19 incident infection*.tw. (196)

20 or/12-18 (297550)

21 11 and 20 (10720)

22 (Acute adj4 (hiv* or ((acquired or human) adj2 immun* adj3 (syndrome* or deficien*)) or human immunedeficien* or human immunodeficien* or human immunideficien*)).tw. (1827)

23 ((recent* or earl* or primary) adj2 (hiv* or ((acquired or human) adj2 immun* adj3 (syndrome* or deficien*)) or human immunedeficien* or human immunodeficien* or human immunideficien*)).tw. (4761)

24 acute retroviral syndrome.tw. (67)

25 incident hiv*.tw. (213)

26 or/22-25 (6498)

27 21 or 26 (14037)

28 5 and 27 (337)

29 human medicine.mc. or human.or. (8193803)

30 28 and 29 (288)

31 remove duplicates from 30 (266)

32 limit 31 to meeting (11)

33 31 not 32 (255)

# PubMed

(Determine*[TIAB]) AND ((((hiv*[TIAB] OR (acquired[TIAB] AND (immune*[TIAB] OR immuno*[TIAB] OR immuni*[TIAB]) AND syndrome*[TIAB]) OR (acquired immun*[TIAB] AND deficien*[TIAB]) OR human[TIAB] immunedeficien*[TIAB] OR human[TIAB] immunodeficien*[TIAB] OR human[TIAB] immunideficien*[TIAB] OR (human[TIAB] AND (immune*[TIAB] OR immuno*[TIAB] OR immuni*[TIAB]) AND deficien*[TIAB])) AND ((acute*[TIAB] AND (infect*[TIAB] OR disease[TIAB])) OR ((recent*[TIAB] OR earl*[TIAB] OR primary[TIAB]) AND (infect*[TIAB] OR disease[TIAB])) OR (immediately[TIAB] AND (post[TIAB] OR after[TIAB] OR following[TIAB]) AND (infect*[TIAB] OR acquisit*[TIAB] OR transmit*[TIAB])) OR (AHI[TIAB] OR AHIs[TIAB] OR PHI[TIAB] OR PHIs[TIAB]) OR (preseroconver*[TIAB] OR pre-seroconver*[TIAB] OR ((recent*[TIAB] OR primary[TIAB] OR new[TIAB] OR newly[TIAB] OR initial*[TIAB] OR preced*[TIAB] OR before[TIAB]) AND (seroconver*[TIAB] OR sero-conver*[TIAB])) OR ((acquisition*[TIAB] OR infection*[TIAB]) AND (seroconver*[TIAB] OR sero-conver*[TIAB]))) OR ((acute[TIAB] OR initial[TIAB] OR ramp-up[TIAB]) AND (phase*[TIAB] OR period*[TIAB] OR stage*[TIAB])) OR (newly[TIAB] AND infected[TIAB]) OR (incident infection*[TIAB])) OR ((Acute[TIAB] OR recent*[TIAB] OR earl*[TIAB] OR primary[TIAB]) AND (hiv*[TIAB] OR ((acquired[TIAB] OR human[TIAB]) AND (immune*[TIAB] OR immuno*[TIAB] OR immuni*[TIAB]) AND (syndrome*[TIAB] OR deficien*[TIAB])) OR human[TIAB] immunedeficien*[TIAB] OR human[TIAB] immunodeficien*[TIAB] OR human[TIAB] immunideficien*[TIAB])) OR ("acute retroviral syndrome"[TIAB]) OR (incident hiv*[TIAB]))) AND ((publisher[sb] NOT pmcbook)))

=3

# African Index Medicus

Search on : Hiv OR (acquired AND immunodeficiency) OR (acquired AND immunedeficiency) OR (acquired AND immuno) OR (acquired AND immune) OR (human AND immunodeficiency) OR (human AND immunedeficiency) OR (human AND immuno) OR (human AND immune) [Key Word] and acute [Key Word] and determine [Key Word]

References found : 0

# LILACS

Via <http://search.bvsalud.org/portal/>

(tw:(Hiv* OR "acquired immunodeficiency" OR "acquired immunedeficiency" OR "acquired immunideficiency" "acquired immuno deficiency" OR "acquired immune deficiency" OR "human immunodeficiency" OR "human immunedeficiency" OR "human immunideficiency" OR "human immune deficiency" OR "human immune deficiency")) AND (tw:(acute OR preseroconversion OR "pre-seroconversion" OR "newly infected" OR ahi OR ahis OR PHI OR PHIs OR "incident infection")) AND (tw:(determine*))

=2
