# Supplementary material for: Evaluation of a Rapid Point of Care Test for Detecting Acute and Established HIV Infection, and Examining the Role of Study Quality on Diagnostic Accuracy: A Bayesian Meta-Analysis
Source: PLoS One. 2016 Feb 18;11(2):e0149592. doi: 10.1371/journal.pone.0149592 (PMC4758636; doi:10.1371/journal.pone.0149592)
Supplement: S1 Table — (DOC) [file pone.0149592.s003.doc]

**Table 1: Demographic characteristics of included studies**

| **Authors** | **Year** | **Country** | **Study Objective** | **Study Design** | **High or Low risk population** | **Reference Standard(s) used** | **Patient Population (general characteristics)** | **Specimen** | **Total Sample Size (n=)** |
| --- | --- | --- | --- | --- | --- | --- | --- | --- | --- |
| Marieke Brauer, et al | 2013 | South Africa | To compare the performance of the Determine Combo test with automated fourth generation HIV ELISAs already in use | Case-control | High risk | 4th generation ELISA followed by confirmatory testing with another fourth generation assay. Seroconversion panel: antibody assay followed by NAAT and p24 assay. | Serum specimens that had been submitted for routine HIV serology testing, plus seroconversion specimens from the South African National Blood Service | serum | 79 |
| Damian P. Conway, et al | 2014 | Australia | To gain a better understanding of the potential of the Determine Combo test for use as a point of care screening assay | Cross-sectional | High risk | 4th generation ELISA, followed by confirmatory antibody testing, HIV p24 assay, and Western Blot | Free access publicly funded sexual health clinics with high caseloads of MSM | whole blood | 3190 |
| Christopher D. Pilcher, et al | 2013 | United States | To determine how use of newer tests might perform given their variable ability to detect acute infections, and how new tests might influence the performance of HIV testing programs in San Francisco. | Case-control | High risk | Initial testing with 1st generation, 3rd generation, or rapid antibody tests, followed by confirmatory antibody testing and RNA. | Stored remnants of blood plasma from STD clinic populations, MSM, Non-occupational post-exposure prophylaxis, partner services testing population, sex worker population, UCSF options study (suspicion of having acute infection) | plasma | 138 |
| Pragna Patel, et al | 2012 | United States | To evaluate the sensitivity for early HIV infection of several rapid tests and third and fourth generation assays compared with NAAT. | Case-control | Unclear | 1st, 2nd, or 3rd generation antibody test or rapid test, followed by NAAT. | Patients from STD clinics, public health clinics, and MSM. | plasma | 33 |
|  |  |  |  |  |  |  | Blinded specimen panel including AHI, Ab-positive and HIV-negative specimens | plasma | 114 |
| Nora E. Rosenberg, et al | 2012 | Malawi | To assess the antigen portion of Determine Combo test to detect persons with acute HIV infection. Also to assess the antibody portion of the test against a standard rapid test antibody algorithm. | Cross-sectional | High risk | Rapid antibody test followed by NAAT. | Patients presenting for HIV testing in HIV testing and counselling centre, patients with negative or discordant HIV rapid test results (at high risk for acute) in STI clinic | whole blood | 1009 |
| Juliette Pavie, et al | 2010 | France | French agency for health product safety mandated that a real-time comparison of five approved rapid tests be carried out on samples from patients with documented HIV infection. Compared fingerstick with whole blood. | Case-control | High (HIV positive) | Western blot (previously confirmed) | Adults with documented HIV infection and HIV seronegative volunteers | whole blood | 220 |
| William Kilembe, et al | 2012 | Zambia and Rwanda | To present results generated by testing specimen panels from adults with or at risk for acquiring HIV infection, and a panel of serially diluted p24 antigen positive controls | Case-control | High | Rapid antibody test, followed by p24 antigen assay, unclear as to whether or not this was followed up with NAAT. | Specimen panels from adults with or at risk for acquiring HIV infection, and a panel of serially diluted p24 antigen postitive controls | plasma | 52 (acute and recent infections) |
| Silvia Faraoni, et al | 2013 | Italy | To establish Determine Combo test performance in acute HIV infection (AHI) in a setting at low HIV-1 prevalence | Case control | Low | Antibody test and Western blot, followed by a PCR-based assay. | Serum samples from patients with AHI, controls from those (HIV negative) seeking HIV testing | serum | 141 |
| Vani Chetty, et al | 2012 | South Africa | To evaluate Determine Combo test against two third generation tests for early detection of HIV infection in pregnancy | Cross-sectional | High | Rapid antibody test | Pregnant women participating in a HIV incidence cohort study who tested negative at baseline | plasma | 253 |
| Julie Fox, et al | 2011 | United Kingdom | To investigate the ability of the Determine Combo test to detect p24 antigen in samples identified as p24-positive using standard of care fourth generation assays. | Case-control | Not reported | 4th generation assay | Stored serum samples following standard laboratory testing | serum | 36 |
| Silvina Masciotra, et al | 2013 | United States | To evaluate the ability of the Determine Combo test to detect acute/early HIV-1 infections and HIV-2 antibody in well-characterized plasma specimens | Case-control | unclear | 4th generation assay | HIV-1 seroconverters previously used to evaluate FDA-approved assays | plasma | 230 |
| Joanne D. Stekler, et al | 2013 | United States | To compare the abilities of POC tests to detect early infection in real time | Cross-sectional | High | 3rd and 4th generation assays, followed by RNA testing | Men and transgender persons reporting sex with men, considered to be at high risk, plus those with suspected/confirmed acute diagnosis | whole blood | Unclear - 25 HIV positive specimens were tested |
| G. Beelaert, and K. Fransen | 2010 | Belgium | To evaluate the Determine fourth generation Combo test using well-characterized HIV panels and a dilution series of HIV culture supernatants. | Case-control | Not reported | Negative specimens: 4th generation ELISA, confirmatory antibody assay, then p24 antigen assay. Positive specimens: 3rd or 4th generation ELISA, confirmation LIA. | Well-characterized archived HIV-postive/negative serum/plasma samples; fresh sepcimens, serum, plasma, and whole blood; supernantants from cultures of different HIV groups and subtypes; p24 antigen control | serum, plasma, and whole blood | 436 |
| Clifford B. Jones, et al | 2012 | United Kingdom | To compare the Determine Combo test with the Abbott Architect fourth generation laboratory assay and the Determine third generation test, in high-risk patients attending a UK genitourinary clinic | Cross-sectional | High | 4th generation ELISA | MSM, persons from HIV endemic areas, injection drug users, or partners of persons in these groups | whole blood | 985 |
| Syria Laperche, et al | 2012 | France | To evaluate the Determine Combo in cell-derived supernatant samples of HIV-1 and HIV-2 subtypes, and in archived plasma specimens | Case-control | Not reported | p24 antigen assay | Cell-derived supernatant samples of HIV-1 and HIV-2 subtypes, and in archived plasma specimens from individuals with acute-phase HIV-1 infection | serum and plasma (supernatant samples) | 119 |
|  |  |  |  |  |  |  |  | plasma (archived samples) | 20 |
| Kapila Bhowan, et al | 2011 | South Africa | To assess the performance of the Determine Combo test against third generation rapid tests in antenatal and post-partum women | Cross-sectional | High | 3rd generation rapid test, discordant results followed by 4th generation testing | Antenatal and early post-partum women without documented HIV status | plasma | 1019 |
|  |  |  |  |  |  |  |  | whole blood | 380 |
| Yen T. Duong, et al | 2014 | Swaziland | To evaluate the performance of the Determine Combo test in detecting acute infections in the Swaziland Incidence Measurement Survey (SHIMS), compared to NAAT results. | Cross-sectional | High | NAAT for Ag-/ Ab+ and Ag- /Ab- specimens, rapid testing for Ab+ (Unigold) confirmed by EIA/NAAT if negative | Adults 18-49 years old, identified through a a nationally representative, household-based cross-sectional survey | whole blood | 18 172 |
