# Supplementary material for: Evaluation of a Rapid Point of Care Test for Detecting Acute and Established HIV Infection, and Examining the Role of Study Quality on Diagnostic Accuracy: A Bayesian Meta-Analysis
Source: PLoS One. 2016 Feb 18;11(2):e0149592. doi: 10.1371/journal.pone.0149592 (PMC4758636; doi:10.1371/journal.pone.0149592)
Supplement: S2 Table — (DOC) [file pone.0149592.s004.doc]

**Table 2:** Raw data included in analysis

| **1^st^ Author** | **Year** | **Study Design** | **Sample** | **Test component** | **Sensitivity** | **Specificity** | **TP** | **FN** | **TN** | **FP** |
| --- | --- | --- | --- | --- | --- | --- | --- | --- | --- | --- |
| **Brauer** | 2013 | case-control | serum/plasma | antibody | 90.7 | 100 | 39 | 4 | 20 | 0 |
| **Brauer** | 2013 | case-control | serum/plasma | antigen | 10 | 100 | 3 | 27 | 20 | 0 |
| **Conway** | 2014 | cross-sectional | whole blood | overall | 87.2 | 99.4 | 34 | 5 | 3133 | 18 |
| **Conway** | 2014 | cross-sectional | whole blood | antibody | 94.4 | 99.6 | 34 | 2 | 3140 | 14 |
| **Conway** | 2014 | cross-sectional | whole blood | antigen | 0 | 99.8 | 0 | 9 | 3175 | 6 |
| **Pilcher** | 2013 | case-control | serum/plasma | overall | 54.4 | 98.8 | 31 | 26 | 80 | 1 |
| **Patel** | 2012 | case-control | serum/plasma | overall | 75.8 | NR | 25 | 8 | NR | NR |
| **Patel** | 2012 | case-control | serum/plasma | overall | 88.1 | 100 | 74 | 10 | 30 | 0 |
| **Rosenberg** | 2012 | cross-sectional | whole blood | antigen | 0 | 98.3 | 0 | 8 | 824 | 14 |
| **Rosenberg** | 2012 | cross-sectional | whole blood | antibody | 99.4 | 99.2 | 162 | 1 | 643 | 5 |
| **Rosenberg** | 2012 | cross-sectional | whole blood | overall | 95.9 | 97.8 | 164 | 7 | 818 | 18 |
| **Pavie** | 2010 | case-control | whole blood | overall | 95.8 | NR | 160 | 7 | NR | NR |
| **Kilembe** | 2012 | case-control | serum/plasma | antigen | 1.9 | NR | 1 | 51 | NR | NR |
| **Faraoni** | 2013 | case-control | serum/plasma | overall | 88.2 | 100 | 15 | 2 | 124 | 0 |
| **Faraoni** | 2013 | case-control | serum/plasma | antigen | 29.4 | 100 | 5 | 12 | 124 | 0 |
| **Faraoni** | 2013 | case-control | serum/plasma | antibody | 58.8 | 100 | 10 | 7 | 124 | 0 |
| **Chetty** | 2012 | cross-sectional | serum/plasma | antibody | 59.4 | 96.9 | 19 | 13 | 31 | 1 |
| **Chetty** | 2012 | cross-sectional | serum/plasma | overall | 94.1 | 96.9 | 208 | 13 | 31 | 1 |
| **Fox** | 2011 | case-control | serum/plasma | antigen | 50 | NR | 18 | 18 | NR | NR |
| **Fox** | 2011 | case-control | serum/plasma | overall | 72.2 | NR | 26 | 10 | NR | NR |
| **Masciotra** | 2013 | case-control | serum/plasma | overall | 92.5 | 97.9 | 124 | 10 | 94 | 2 |
| **Masciotra** | 2013 | case-control | serum/plasma | antigen | 72.2 | 96.2 | 26 | 10 | 75 | 3 |
| **Stekler** | 2013 | cross-sectional | whole blood | overall | 88.0 | 99.7 | 22 | 3 | NR | NR |
| **Beelaert** | 2010 | case-control | serum, plasma, and whole blood | antibody | 100 | NR | 169 | 0 | NR | NR |
| **Beelaert** | 2010 | case-control | serum, plasma, and whole blood | antigen | 86.6 | NR | 58 | 9 | NR | NR |
| **Beelaert** | 2010 | case-control | serum, plasma, and whole blood | overall | NR | 100 | NR | NR | 100 | 0 |
| **Jones** | 2012 | cross-sectional | whole blood | overall | 90.5 | 99.8 | 19 | 2 | NR | NR |
| **Laperche** | 2012 | case-control | serum/plasma | antigen | 21.0 | NR | 25 | 94 | NR | NR |
| **Laperche** | 2012 | case-control | serum/plasma | antigen | 60 | NR | 12 | 8 | NR | NR |
| **Bhowan** | 2011 | cross-sectional | serum/plasma | antibody | 100 | 99.8 | 90 | 0 | 927 | 2 |
| **Bhowan** | 2011 | cross-sectional | whole blood | antibody | 100 | 99.3 | 89 | 0 | 289 | 2 |
| **Duong** | 2014 | Cross-sectional | Whole blood | antigen | 0 | 99.9 | 0 | 13 | 12345 | 12 |

NR = “not reported”; the data could not be extracted from the information available.
